# Result of Potassium-Competitive Acid Blockers in the Treatment of Severe Gastroesophageal Reflux Disease-Associated Esophageal Ulcer

**DOI:** 10.7759/cureus.105634

**Published:** 2026-03-22

**Authors:** Takayoshi Tsubaki, Masami Hashidume, Keimei Kato

**Affiliations:** 1 Department of Internal Medicine, Kimura Hospital, Fukuoka, JPN; 2 Endoscopy Center, Sakai Municipal Mikuni Hospital, Sakai, JPN; 3 Department of Internal Medicine, Sakai Municipal Mikuni Hospital, Sakai, JPN

**Keywords:** acid-suppressive therapy, gastroesophageal reflux disease, h⁺/k⁺-atpase transporter, potassium-competitive acid blockers, vonoprazan

## Abstract

Esophageal ulcers are rare but can become severe and require prompt treatment. Gastroesophageal reflux disease (GERD), one of the causes, is a frequently encountered disease that considerably affects the daily lives of patients; therefore, appropriate management is required. Acid-suppressive therapy is the mainstay of treatment. Here, we report a case in which potassium-competitive acid blocker (P-CAB) therapy was associated with ulcer healing and symptom improvement caused by GERD. An 81-year-old woman was referred for further evaluation after abnormal gastric imaging findings were detected during a routine health checkup. The patient had no remarkable medical history or comorbidities. Upper gastrointestinal endoscopy revealed an esophageal ulcer caused by severe GERD. Acid-suppressive therapy with P-CABs was effective, resulting in the rapid resolution of the ulcer and improvement of reflux-like symptoms. The dose was subsequently reduced as maintenance therapy, and the patient continued to do well without symptom recurrence. As illustrated in the present case, GERD has the potential to progress to esophageal ulceration; therefore, prompt initiation of treatment is necessary. P-CABs may be a useful therapeutic option in similar cases.

## Introduction

Esophageal ulcers are less common than mild erosive esophagitis but may occur in severe gastroesophageal reflux disease (GERD). Other causes besides GERD include the use of nonsteroidal anti-inflammatory drugs, antibiotics, infections, and malignancies [1]. Common clinical symptoms include gastrointestinal bleeding, abdominal pain, and chest pain [1]. GERD is defined as experiencing heartburn or acid regurgitation at least once a week. Although its exact global prevalence is unknown, it is estimated to range from 5% to 25% [2]. Esophageal ulcers can become severe, and prompt treatment is required. Acid-suppressive therapy is the mainstay of treatment for GERD, the most common cause of esophageal ulcers [3]. Potassium-competitive acid blockers (P-CABs), such as vonoprazan, act on the H⁺/K⁺-ATPase transporter present in gastric parietal cells and suppress gastric acid secretion by inhibiting the exchange of H⁺ and K⁺ [4,5]. Compared with conventional proton pump inhibitors (PPIs), P-CABs have several advantages, including a more rapid onset of action, longer duration of acid suppression, and less interindividual variability in acid-suppressive effects [6].

While P-CABs have shown effectiveness in treating erosive esophagitis, there is a scarcity of reports specifically detailing their efficacy in cases of severe GERD-related esophageal ulceration. Here, we report a case of a severe esophageal ulcer secondary to GERD that was incidentally discovered during a routine health checkup and was successfully treated with a P-CAB.

## Case presentation

An 81-year-old woman was referred to our hospital for further evaluation after an irregular margin at the esophagogastric junction was detected during an upper gastrointestinal barium study performed as part of a gastric cancer screening program. She had no remarkable medical or family history and was not receiving regular medical follow-up or any medications.

Physical examination revealed that she was 157.8 cm tall and weighed 49 kg. Her abdomen was flat and soft with no tenderness, and her vital signs were stable. Regarding subjective symptoms, the patient reported a sensation of food sticking and not passing smoothly during meals.

**Figure 1:**
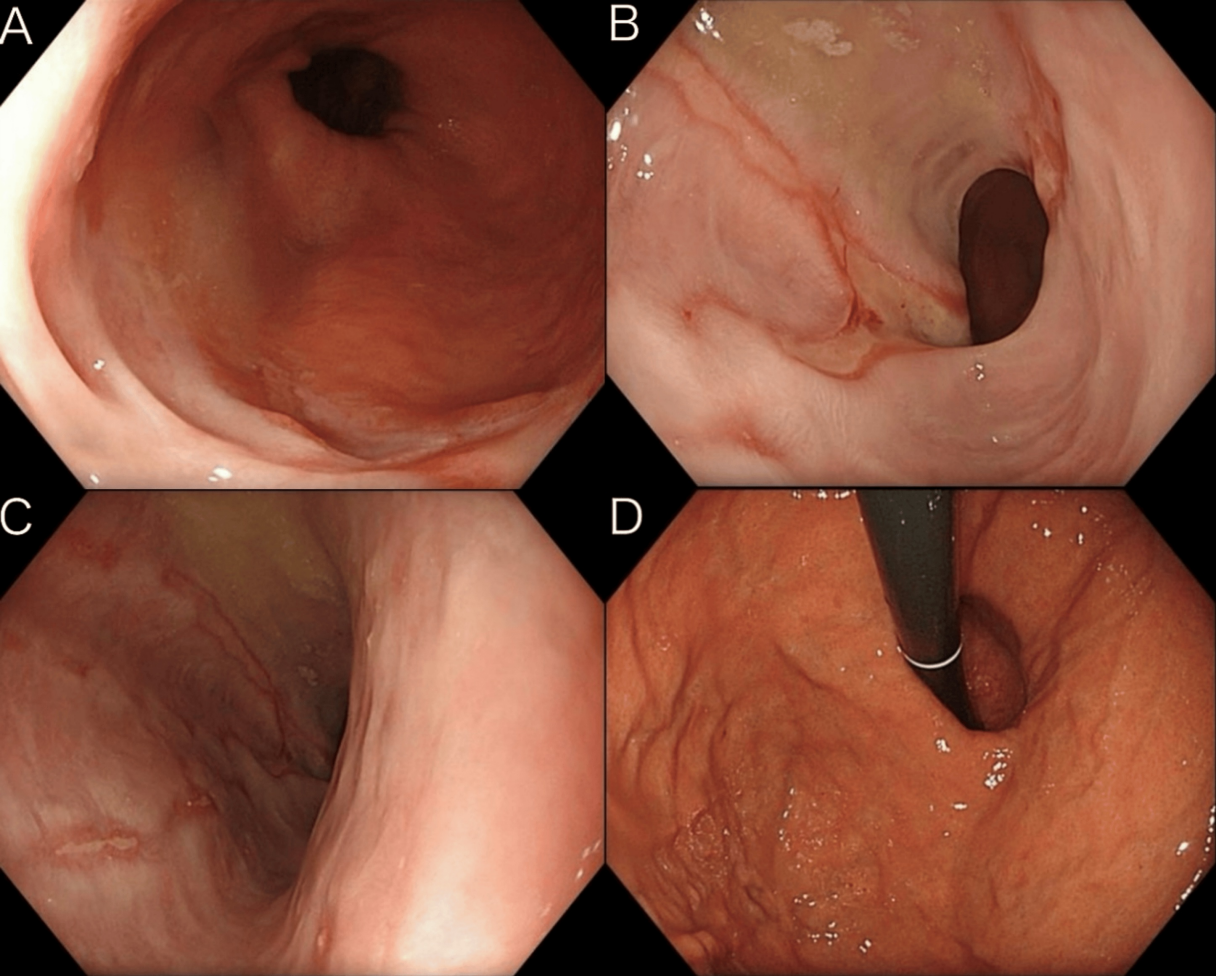
Findings of upper gastrointestinal endoscopy at the initial visit. A: Mucosa at the esophagogastric junction showing circumferential erythema with partial ulceration. B: Erythematous changes extended proximally, forming broad-based ulcers. C: Small scattered ulcers were observed proximally. D: Findings consistent with a hiatal hernia.

The findings of the upper gastrointestinal endoscopy performed at the initial visit are shown in Figure 1. The esophageal mucosa at the esophagogastric junction exhibited near-circumferential erythema with partial ulceration (Figure 1A). The erythematous changes extended toward the oral side at two sites, each forming a broad-based ulcer (Figure 1B). The ulcers extended proximally in a linear fashion and were observed as scattered small ulcers (Figure 1C). In addition, findings consistent with a hiatal hernia were noted (Figure 1D). The endoscopic observations were in line with Los Angeles Grade D (Table 1, quoted from [7]) reflux esophagitis linked to a hiatal hernia. Although reflux monitoring was not conducted, the clinical and endoscopic evidence strongly indicated GERD. Considering the possibility of ulceration because of malignancy, biopsies were obtained from all three sites. Oral administration of a P-CAB at a dose of 20 mg/day was initiated on the same day.

**Table 1:** The Los Angels classification of esophagitis. This table was created based on [7].

Grade	Description
Grade A	One (or more) mucosal break no longer than 5 mm that does not extend between the tops of two mucosal folds
Grade B	One (or more) mucosal break more than 5 mm long that does not extend between the tops of two mucosal folds
Grade C	One (or more) mucosal break that is continuous between the tops of two or more mucosal folds but which involves less than 75% of the circumference
Grade D	One (or more) mucosal break which involves at least 75% of the esophageal circumference

**Figure 2:**
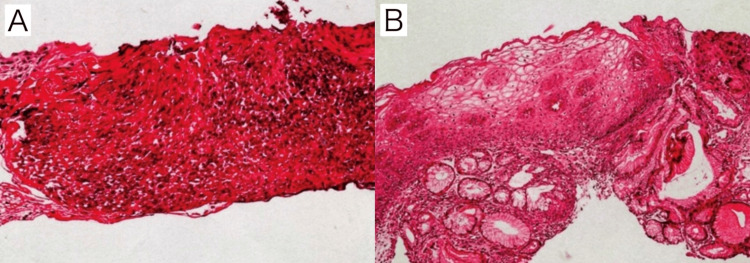
Histopathological findings. A: Irregular thickening of the stratified squamous epithelium was observed; however, the layered architecture was preserved, and no atypia or pleomorphism was noted. B: The specimen included gastric foveolar epithelium with cardiac-type glands, and the stroma was congested and edematous with infiltration of inflammatory cells, including neutrophils, lymphocytes, and plasma cells; however, no findings suggestive of malignancy were identified.

At the follow-up visit 14 days later, her reflux-like symptoms had resolved. The biopsy results from the initial endoscopy were as follows: irregular thickening of the stratified squamous epithelium was observed; however, the layered architecture was preserved, and no atypia or pleomorphism was noted (Figure 2A). Inflammatory cell infiltration was also present; however, no findings suggestive of malignancy were observed (Figure 2B). Therefore, there was no change in the diagnosis.

**Figure 3:**
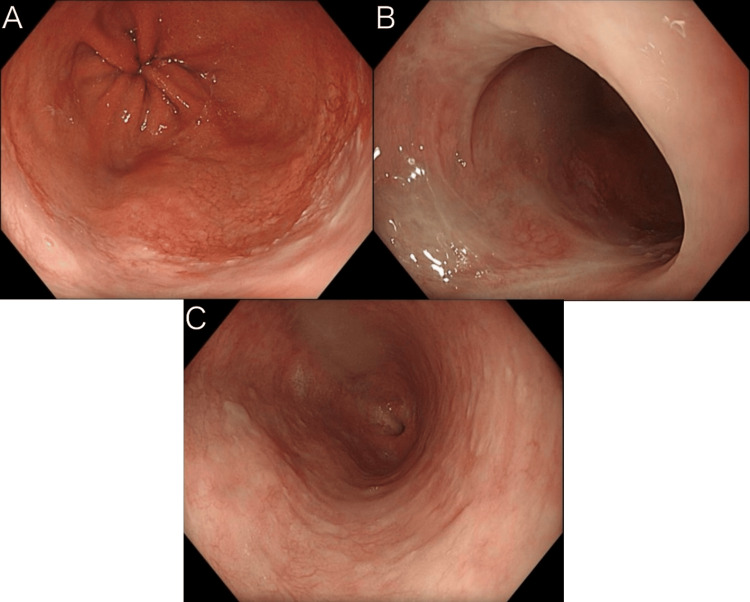
Findings of upper gastrointestinal endoscopy three months after the initial visit. A: Ulcer at the esophagogastric junction had resolved. B: Broad-based ulcers that had extended proximally had also disappeared. C: Scattered small ulcers further proximally had likewise resolved.

Upper gastrointestinal endoscopy was performed for follow-up three months after the initial visit. During this period, her reflux-like chest symptoms remained well controlled, and no remarkable subjective symptoms were noted. The endoscopic findings are shown in Figure 3. The esophagogastric junction showed some residual redness, but the ulcer had disappeared (Figure 3A). All ulcers, including those that had spread extensively in the two areas and those progressing toward the oral side, were completely resolved (Figure 3B, 3C). Endoscopic examination confirmed improvement in reflux esophagitis and esophageal ulcers.

**Figure 4:**
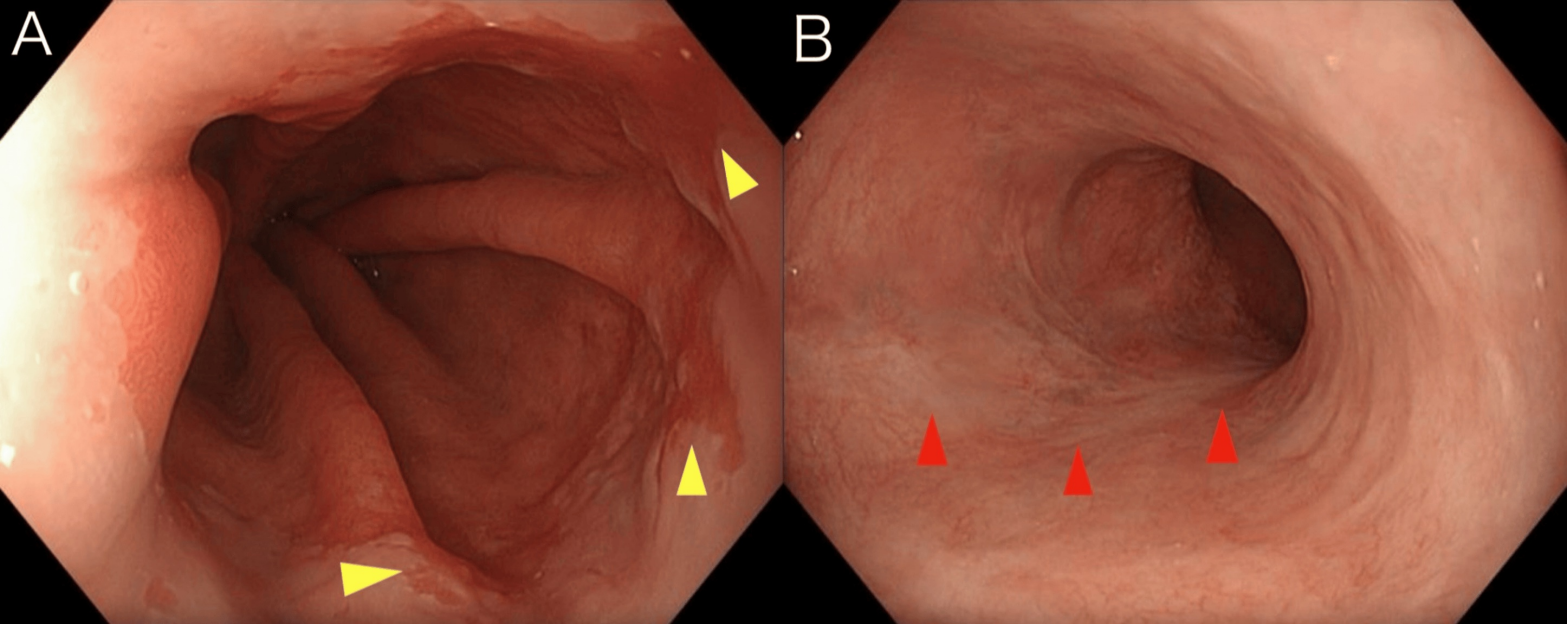
Endoscopic findings 15 months after the initial visit. A: Improvement in gastroesophageal reflux disease was maintained (indicated by the yellow arrows). B: Sustained disappearance of the ulcers (indicated by the red arrows).

P-CAB therapy was continued at a dose of 20 mg/day; however, as the patient’s subjective symptoms remained stable thereafter, the dose was reduced to 10 mg/day at a visit six months after the previous endoscopy (nine months after the initial visit). A follow-up upper gastrointestinal endoscopy was performed an additional six months later (15 months after the initial visit). As in the previous examination, sustained disappearance of the ulcers and continued improvement in GERD were observed (Figures 4A, 4B).

The patient continued oral P-CAB therapy at 10 mg/day, and no symptom recurrence has been observed to date.

## Discussion

Esophageal ulcers are a relatively rare condition, and according to Cohen et al., the prevalence rate was 0.49% among the 20,382 patients who underwent endoscopy [1]. P-CABs are currently used as acid-suppressive agents to treat gastric ulcers, duodenal ulcers, and GERD. Unlike conventional PPIs, P-CABs exert their effects by acting on the H⁺/K⁺-ATPase transporter in gastric parietal cells and inhibiting the exchange of H⁺ and K⁺ [4]. They have several advantages, including a rapid onset of action, good stability under acidic conditions, and a prolonged duration of plasma concentration maintenance [8]. P-CAB has the potential to meet the needs of patients undergoing treatment for acid-related diseases.

In the present case, treatment with a P-CAB at a dose of 20 mg/day was initiated for an esophageal ulcer secondary to severe GERD, and prompt resolution of subjective symptoms was observed. Upper gastrointestinal endoscopy performed three months later demonstrated the complete disappearance of the ulcer, confirming clinical improvement. As maintenance therapy, oral administration of a P-CAB (10 mg/day) was continued for six months, during which no evidence of recurrence was observed. A favorable therapeutic outcome was achieved, which is consistent with the findings of previous reports. The GERD in this case was considered to be caused by a severe hiatal hernia; however, other possible mechanisms include relaxation of the lower esophageal sphincter, increased intra-abdominal pressure, and decreased lower esophageal sphincter pressure [9]. Esophageal reflux monitoring (pH or impedance pH) and PPI testing are useful for these diagnoses [9], but they were not performed in this case, and PPIs were not used at the start of treatment. As no prior comparison with PPIs was conducted, it cannot be said that P-CAB was significantly more effective than PPIs in this case. However, the ulcer was severe on endoscopic examination, as reported by Oshima and Miwa [8]. As P-CAB has several characteristics that distinguish it from PPIs, we decided to try it as the initial treatment. If a patient is resistant to PPIs or has not responded to PPI therapy in the past, P-CAB may be considered as a first-line treatment.

In the present case, treatment was initiated at a dose of 20 mg/day, resulting in the rapid resolution of both the esophageal ulcers and GERD. Even with maintenance therapy at 10 mg/day, sustained disappearance of the ulcer and continued resolution of GERD symptoms were achieved, demonstrating the marked efficacy of P-CAB therapy. No notable adverse events were observed, and long-term continuation of therapy was possible. GERD is a disease commonly encountered in daily clinical practice and can markedly impair quality of life depending on symptom severity. In some cases, it may progress to esophageal ulceration, as observed in the present case. GERD requires prompt and long-term management, and P-CABs are considered highly effective for this purpose.

## Conclusions

GERD is a common condition, and many patients experience symptoms, such as heartburn or acid regurgitation. Prompt treatment is required because the disease can progress to severe complications, such as esophageal ulceration. Acid-suppressive therapy was effective in the present case. Although additional comparative studies are needed to determine whether P-CABs offer advantages over standard PPI therapy in cases of severe GERD-related ulcers, in this case, P-CAB therapy was associated with ulcer healing and sustained symptom control. Early initiation of therapy, before progression to severe disease, is important to preserve patients’ quality of life.
